# Lymphocyte depletion and repopulation after chemotherapy for primary breast cancer

**DOI:** 10.1186/s13058-015-0669-x

**Published:** 2016-01-26

**Authors:** Rashmi Verma, Ruth E. Foster, Kieran Horgan, Katherine Mounsey, Helen Nixon, Natuley Smalle, Thomas A. Hughes, Clive RD. Carter

**Affiliations:** School of Medicine, University of Leeds, Leeds, UK; Department of Breast Surgery, Leeds Teaching Hospitals NHS Trust, Leeds, UK; Department of Transplant Immunology, Leeds Teaching Hospitals NHS Trust, Leeds, UK; Clinical Immunology Laboratories, Leeds Teaching Hospitals NHS Trust, Leeds, UK

**Keywords:** Breast cancer, Chemotherapy, Smoking, B lymphocytes, Memory B cells

## Abstract

**Background:**

Approximately 30 % of breast cancer patients receive chemotherapy, yet little is known about influences of current regimens on circulating lymphocyte levels and phenotypes. Similarly, clinico-pathological factors that modify these influences, and implications for future immune health remain mainly unexplored.

**Methods:**

We used flow-cytometry to assess circulating lymphocyte levels and phenotypes in 88 primary breast cancer patients before chemotherapy and at time-points from 2 weeks to 9 months after chemotherapy completion. We examined circulating titres of antibodies against pneumococcal and tetanus antigens using ELISAs.

**Results:**

Levels of B, T and NK cells were significantly reduced 2 weeks after chemotherapy (*p* < 0.001). B cells demonstrated particularly dramatic depletion, falling to 5.4 % of pre-chemotherapy levels. Levels of all cells recovered to some extent, although B and CD4^+^ T cells remained significantly depleted even 9 months post-chemotherapy (*p* < 0.001). Phenotypes of repopulating B and CD4^+^ T cells were significantly different from, and showed no sign of returning to pre-chemotherapy profiles. Repopulating B cells were highly depleted in memory cells, with proportions of memory cells falling from 38 % to 10 % (*p* < 0.001). Conversely, repopulating CD4^+^ T cells were enriched in memory cells, which increased from 63 % to 75 % (*p* < 0.001). Differences in chemotherapy regimen and patient smoking were associated with significant differences in depletion extent or repopulation dynamics. Titres of anti-pneumococcal and anti-tetanus antibodies were both significantly reduced post-chemotherapy and did not recover during the study (*p* < 0.001).

**Conclusion:**

Breast cancer chemotherapy is associated with long-term changes in immune parameters that should be considered during clinical management.

**Electronic supplementary material:**

The online version of this article (doi:10.1186/s13058-015-0669-x) contains supplementary material, which is available to authorized users.

## Background

Breast cancer is the most common malignancy in women and causes more than half a million deaths annually worldwide [1]. Typical treatment is surgical tumour resection, usually combined with endocrine therapy, biologics, radiotherapy, and/or cytotoxic chemotherapy. Chemotherapy is a component of therapy in ~30 % of cases [2], and is recommended when tumours display poor prognosis features, including nodal involvement, large size, high grade, and/or lack of expression of estrogen and progesterone receptors [3]. Current chemotherapy regimens for primary disease include anthracycline-based protocols and sequential use of anthracyclines and taxanes, and these give substantial reductions in metastatic recurrence rates and increases in overall survival [4, 5]. However, chemotherapy is also associated with wide ranging adverse effects on non-target tissues, including substantial impacts on the immune system. Neutropenia is often regarded as the most serious haematological toxicity and can be associated with infections that may force chemotherapy dose reduction and/or delays that may compromise treatment [6, 7]. Neutrophil levels are known to recover after therapy with appropriate management and this transient neutropenia is not thought to have common persistent consequences. Chemotherapy also affects the adaptive immune system, and by contrast, there is evidence these effects may cause more long-lived changes to immunity, although studies in the context of modern chemotherapy regimens and particularly with respect to B lymphocytes are lacking.

Many studies have reported effects of chemotherapy on lymphocytes in breast cancer patients during the therapy itself or up to 3 months after the last chemotherapy cycle, with a consensus that chemotherapy reduces circulating lymphocyte levels [8–12]. Lymphopenia shortly following chemotherapy for many other cancers is also well established [13, 14]. Much less is known about whether, when and how lymphocyte populations recover in the longer-term, and what is known is often conflicting. For example, significantly depressed T and B cell numbers as long as 12 months following completion of chemotherapy have been reported [15], while others have found all lymphocyte populations except CD4^+^ T cells to recover to pre-treatment levels at the same time-point, even after a particularly dose intense chemotherapy regimen [16]. A key issue is that extrapolating currently relevant conclusions from this aging literature may be impossible as modern chemotherapy regimens differ substantially from those in much of the literature. Nevertheless, common themes that have some support in more modern literature are discernable. With respect to T cells, it appears that CD8^+^ T cell levels recover more quickly after chemotherapy than CD4^+^ T cells [9–11, 13, 16] and that repopulating cells comprise a reduced proportion of naïve cells, and an increased memory component [16, 17]. With respect to B cells, there is a paucity of published data beyond the generic observation that B cell levels are reduced post-chemotherapy. The phenotype of repopulating B cells remains essentially unknown, and there is no published understanding of how B cell repopulation might impact on subsequent immunity. In this work we have analysed lymphocyte levels and phenotypes in a cohort of breast cancer patients before, and at various time-points after chemotherapy in an effort to understand the longer-term changes associated with chemotherapy and the clinico-pathological factors that may influence them, the interplay between the various repopulating lymphocyte subtypes, and the potential implications of repopulation for future immune health.

## Methods

### Ethical issues, participants, sample collection

Ethical approval was obtained from Leeds (East) Research Ethics Committee (ref 06/Q1206/217). Patients at Leeds Teaching Hospitals NHS Trust diagnosed between June 2011 and Jan 2012 with operable primary breast cancer and receiving chemotherapy were eligible for the study. Exclusion criteria were: male; aged >75 years; long-term steroid/immunosuppressive drug use; previous breast cancer history; previous chemotherapy in last 10 years. 88 patients were recruited and peripheral blood – two separate 4ml samples taken in an ethylene-diamine-tetra acetic acid vacuette tube (Greiner Bio-one) (for lymphocyte analyses) and a clot activator/gel tube (Greiner Bioone) (for serum separation) – was taken prior to starting chemotherapy and at 2 weeks, 3, 6 and 9 months after the last chemotherapy cycle. In 26 patients, pre-chemotherapy samples were unavailable, and these patients are excluded from analyses relative to pre-chemotherapy levels, and data for pre-chemotherapy time-points are *n* = 62. Extensive clinico-pathological data concerning patients were collected (Table 1). We defined ‘smokers’ as all current smokers (irrespective of number of cigarettes smoked and duration of smoking); ‘non-smokers’ were all ex-smokers and non-smokers. Healthy controls were also recruited (*n* = 17) and peripheral blood taken as above on a single occasion. All participants gave informed written consent.

**Table 1:** Clinico-pathological features of the breast cancer patients included in the study

		number (%) (*n* = 88)
Age	range: 24-74	
	mean: 52	
Smoking	smokers	25 (28)
	non-smokers	63 (72)
Tumour grade	1	2 (2)
	2	37 (42)
	3	49 (56)
Tumour size	<2cm	42 (48)
	2 - 5cm	33 (37)
	> = 5cm	13 (15)
Surgery	breast conservation	53 (60)
	mastectomy	34 (39)
	no surgery	1 (1)
ER/PR status	positive	57 (65)
	negative	31 (35)
Her2 status	positive	16 (18)
	negative	72 (82)
Nodal status	negative	38 (43)
	positive	50 (57)
Chemotherapy	EC/FEC	39 (44)
	EC + TAX (+G-CSF)	44 (50)
	others	6 (7)
	neoadjuvant	18 (20)
	adjuvant	70 (80)
Endocrine	none	31 (35)
	tamoxifen	37 (42)
	aromatase inhibitors	20 (23)
Radiotherapy	yes	73 (83)
	no	15 (17)

### Lymphocyte subset analyses

Absolute numbers and percentages of lymphocyte subsets were determined by flow cytometry using Trucount tubes (Becton Dickinson) and a ‘lyse non-wash’ protocol, according to manufacturer’s instructions. All samples were tested <16h after collection. Briefly, 50μl whole blood was incubated with 15μl multitest reagents (CD3/CD8/CD45/CD4 and CD3/CD16-CD56/CD3/CD45; Becton Dickinson) (15min, dark, room temperature). Lysing buffer (450μl) was added and samples were incubated for a further 15min prior to data acquisition using multiset software on a FACSCalibur flow cytometer (Becton Dickinson). Using this method, the following lymphocyte subsets were measured: T (CD45^+^ CD3^+^); B (CD45^+^ CD19^+^); helper T (CD45^+^ CD3^+^ CD4^+^); cytotoxic T (CD45^+^ CD3^+^ CD8^+^); NK (CD45^+^ CD3^−^ CD56^+^ and/or CD16^+^). For more detailed phenotyping 100μl blood was incubated with mixtures of antibodies at appropriate concentrations (outlined below) (20min, room temperature, dark). Samples were subsequently treated with 3ml lysis solution and were washed twice in 1 % FBS in PBS. Cells were resuspeded in 400μl 5 % formaldehyde in PBS and 25,000 events were acquired on a FACSCanto flow cytometer before analysis in CellQuest (Becton Dickinson). Positive expression was defined using negative control antibody staining to define the negative/positive cut offs, and these were consistently applied in all samples. Hi/low cut offs were arbitrary, but consistently applied in all samples. Antibodies used were CD19-PerCP (clone SJ25C1); CD27-FITC/Pe (M-T271); IgD-FITC (IA6-2); CD24-Pe (ML5); CD38-APC (HB7), CD3PerCP (SK7); CD4-APC (SK3); CD56^RA^-Pe (HI100), CD45^RO^-FITC (UCHL-1), CD62L-FITC (Dreg56) CD31-Pe (L133.1). Using this method the following lymphocyte subtypes were quantified: naïve B (CD19^+^ CD27^−^ IgD^+^); non-switched memory B (CD19^+^ CD27^+^ IgD^+^); switched memory B (CD19^+^ CD27^+^ IgD^−^); transitional B (CD19^+^ CD24^hi^ CD38^hi^); naïve T (CD4^+^ RA^+^ RO^−^ or CD4^+^ RA^+^ CD62L^+^); memory T (CD4^+^ RA^−^ RO^+^); and recent thymic emigrants (CD4^+^ CD45^RA+^ CD31^+^).

### Serum separation and determination of antibody titres

Serum was separated from clot activator/gel blood sample tubes after centrifugation (~500g, 20min, room temperature), and was stored at -20 °C. Tetanus and pneumococcal antibody titres and thresholds for “suboptimal” and “inadequate” levels were determined by enzyme immunoassay kits (The Binding Site) following the manufacturer’s protocols.

### Statistical analysis

Analyses were performed using SPSSv17 (IBM). Tests used included Mann-Whitney U, Wilcoxan signed rank, Kruskal Wallis, related samples Friedman’s 2 way ANOVA, Pearson Chi square test, Linear-by-linear Association and Spearman’s correlation. Values for *p* < 0.05 were considered statistically significant.

## Results

### Lymphocyte subtypes show differential depletion and recovery after chemotherapy

**Fig 1:**
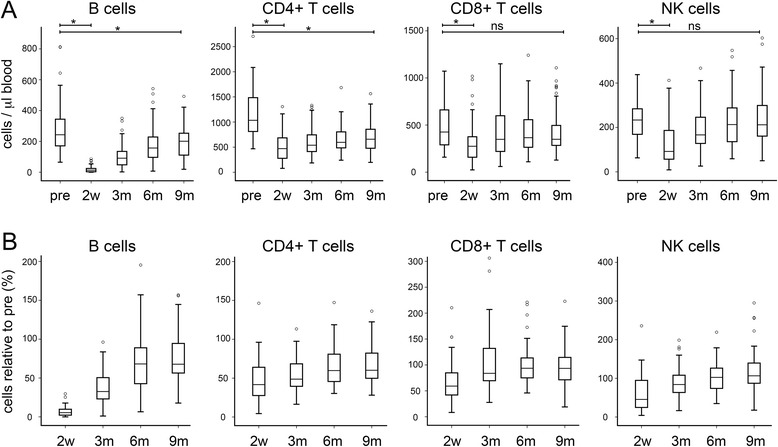
Lymphocyte subtypes show differential depletion and recovery after chemotherapy. Absolute numbers of the lymphocyte subgroups shown were determined by multi-parameter flow cytometry on peripheral blood samples taken from 88 breast cancer patients either before chemotherapy (pre) or at various time-points after the end of chemotherapy (2 weeks [2w]; 3, 6 or 9 months [3m, 6m, 9m]). Data are shown as absolute counts (**a**) or relative to the matched pre-chemotherapy level (**b**). Boxes represent 50 % of the data, with medians (lines), interquartile ranges (whiskers) and individual outliers (circles). * *p* < 0.001 (shown for selected comparisons only); ns ‘not significant’

In order to understand better the effects of chemotherapy on the adaptive immune system, we conducted a longitudinal investigation of lymphocyte levels and phenotypes before and at time-points from 2 weeks to 9 months after the end of chemotherapy. 88 patients treated with chemotherapy for primary breast cancer were included and extensive clinico-pathological data concerning the patients were collected (Table 1). Levels of peripheral blood B cells, CD4^+^ T cells, CD8^+^ T cells, and NK cells were determined and are presented as absolute levels (Fig. 1a), and as levels relative to the matched pre-chemotherapy level for each patient (Fig. 1b).

Levels of each lymphocyte subtype prior to chemotherapy were within the normal range in all cases, with a relatively large degree of variation across the cohort as expected within any population; these levels in patients did not significantly differ from those in healthy subjects of a similar age range (Additional file 1: Table S1). 2 weeks after the end of chemotherapy, significant depletions of all 4 lymphocyte subtypes were noted as compared to pre-chemotherapy levels (*p* < 0.001). B cells demonstrated a particularly dramatic depletion, falling to a median of 5.4 % of pre-chemotherapy levels. In the majority of patients (55.7 %), B cells were reduced to <2 % of their original levels, while 8 individuals showed absolute B cell counts as low as <1 cell/μl. Levels of each lymphocyte subtype demonstrated gradual recovery during the study, until by 9 months post-chemotherapy levels of CD8^+^ T and NK cells were close to, and no longer significantly different from pre-chemotherapy levels. However, there was only partial recovery of B and CD4^+^ T cells after 9 months (reaching medians of only 69 % and 60 % of initial levels respectively) and these levels remained significantly different from pre-chemotherapy levels at this time-point (*p* < 0.001). In addition, in both of these cell types there was no evidence of continued recovery from 6 months (68 % and 60 % of initial levels) to 9 months.

The extents of depletion relative to pre-chemotherapy levels for each subtype were highly correlated (Spearman’s coefficients 0.56-0.84; *p* < 0.001), with particularly strong relationships within the B and T cells (coefficients all >0.74), demonstrating that the greatest or least depletion of each subtype typically occurred in the same individuals. Reconstitution relative to initial levels for the different subtypes was, by contrast, only weakly correlated between B, CD4^+^ T and NK cells, with coefficients of 0.267-0.383 (*p* < 0.05) at 3 and 6 months post-chemotherapy and no significant relationships at 9 months, suggesting that recovery of these cell types was broadly independent. Exceptions to this were between CD8^+^ and CD4^+^ T cells, where moderately strong relationships were maintained at every recovery time-point (coefficients 0.48-0.72; *p* < 0.001), and between CD8^+^ T and NK cells, which behaved similarly (coefficients 0.47-0.52; *p* < 0.001), indicating that recoveries of these pairs of cell populations may be functionally related.

**Fig 2:**
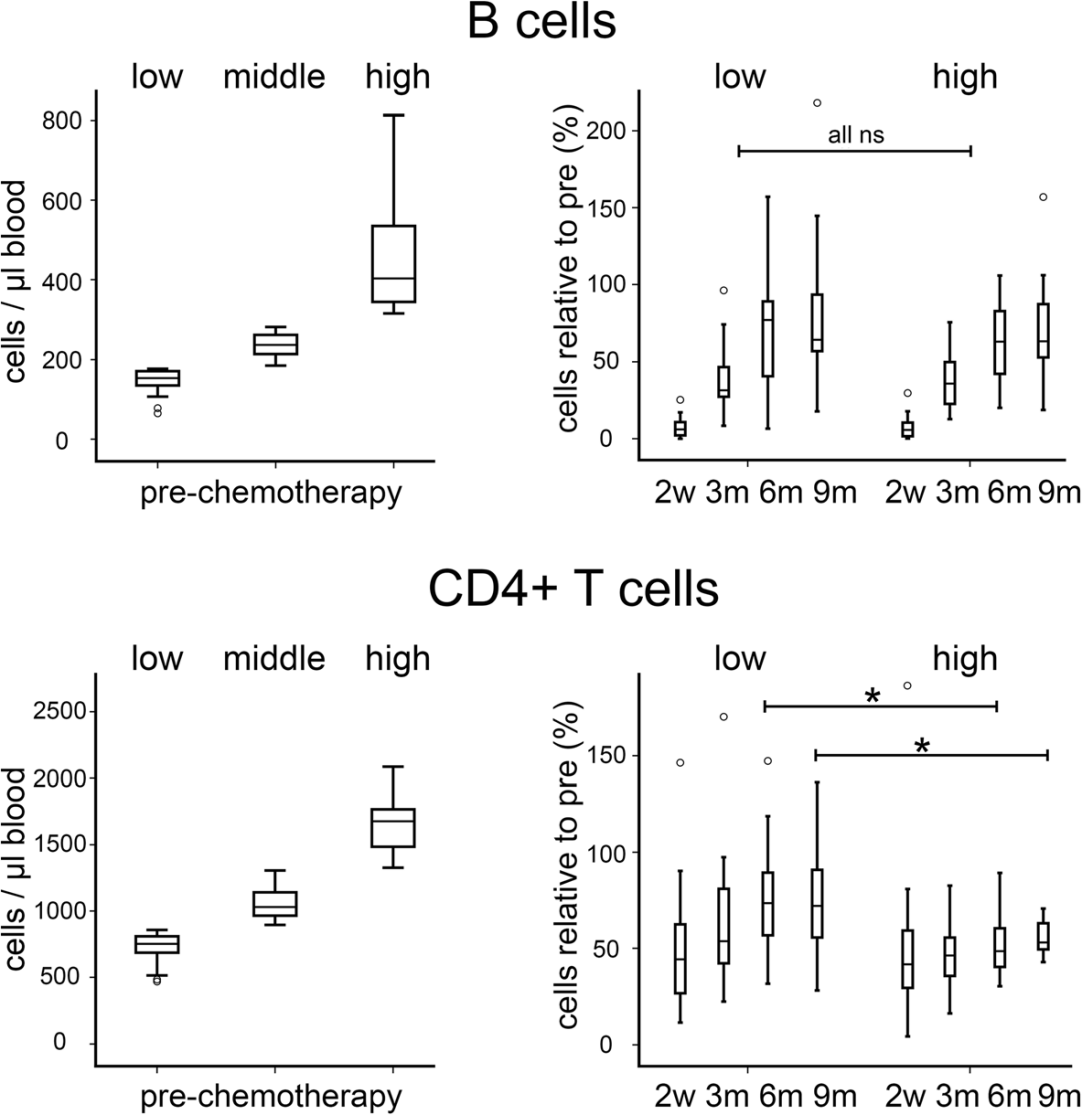
B cell recovery is not influenced by pre-chemotherapy levels of B cells while CD4+ T cell recovery is significantly impaired in patients with the highest pre-chemotherapy CD4+ T cell levels. Patients were divided into three tertiles based on pre-chemotherapy absolute counts of B cells (top left) or CD4+ T cells (bottom left). Relative levels of these cell types at time-points after the end of chemotherapy (2 weeks [2w]; 3, 6 or 9 months [3m, 6m, 9m]) are shown for the upper and lower tertiles (right panels) and were compared pairwise at each time-point (* *p* < 0.02). Boxes represent 50 % of the data, with medians (lines), interquartile ranges (whiskers) and individual outliers (circles)

Subsequently, we focused on the B and CD4^+^ T subtypes, as these remained significantly depressed throughout the study. We investigated whether their degrees of depletion or kinetics of reconstitution were related to pre-chemotherapy levels, by comparing depletion and reconstitution in patients within the top or bottom tertiles of pre-chemotherapy levels (Fig. 2). For B cells, patients with high and low initial levels suffered similar degrees of depletion (to 5.7 % and 6 %; *p* = 0.93) and levels recovered at similar rates (for example, to 63 % and 64 % at 9 months; *p* = 0.71); there was no evidence that pre-chemotherapy levels impacted on relative depletion and reconstitution at any time-point. For CD4^+^ T cells, patients with high and low initial levels similarly showed no difference in depletion (to 42 % and 44 %; *p* = 0.62). However, reconstitution of these cells differed significantly between the groups (*p* < 0.02). Patients with the lowest initial levels recovered CD4^+^ T cells to 73 % of these levels after 6 months, although no further recovery was seen at 9 months (72 %). This contrasts with patients with the highest initial levels, where levels reached only 48 % and 53 % of these levels at 6 and 9 months respectively. This reduced relative recovery was not solely a consequence of presenting data as a proportion of the higher initial levels in this group, as slower recovery was also evident in a comparison of increases in absolute counts. Patients in the upper tertile of initial levels showed smaller numerical increases in absolute counts of CD4^+^ T cells than those seen in patients in the lower tertile at all three recovery time-points.

### Chemotherapy increases proportions of naïve and decreases proportions of memory B cells

**Fig 3:**
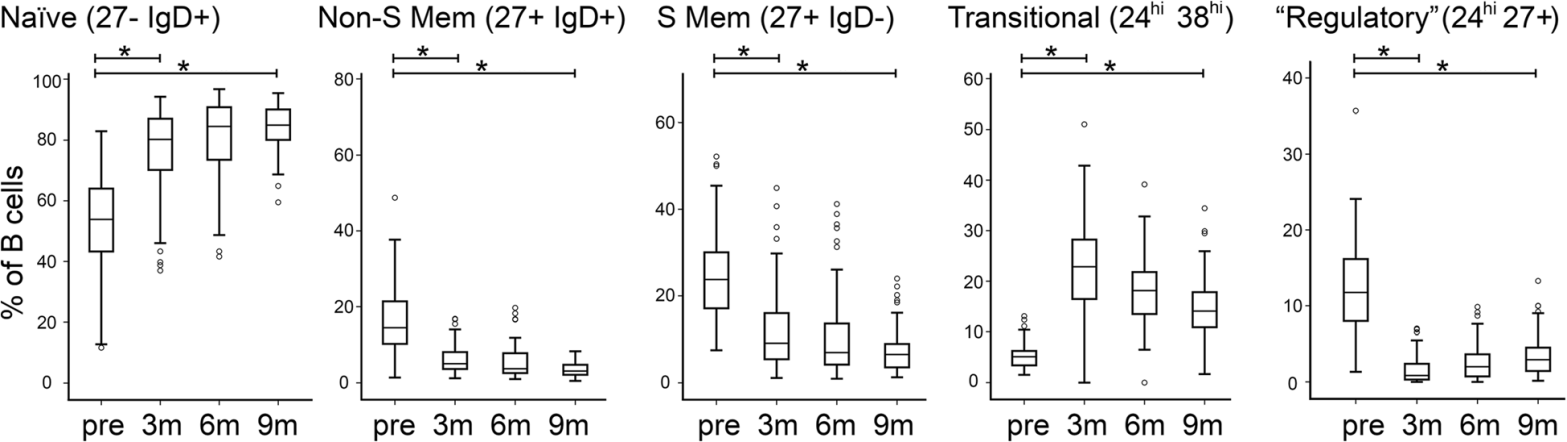
After chemotherapy B cells comprise more naïve cells and fewer memory cells, a change that does not show a trend towards normalising even 9 months after chemotherapy. B cell subtypes were quantified by multi-parameter flow cytometry and their numbers are presented as the proportion of the total B cell pool. Data are shown for samples taken pre-chemotherapy (pre) and 3, 6, and 9 months (3m, 6m, 9m) after the end of chemotherapy. Boxes represent 50 % of the data, with medians (lines), interquartile ranges (whiskers) and individual outliers (circles). * *p* < 0.001

We next investigated the effect of chemotherapy on B phenotypes in more detail. CD19^+^ B cells were analysed as: naïve (CD27^−^ IgD^+^); non-switched memory (CD27^+^ IgD^+^); switched memory (CD27^+^ IgD^−^), transitional (CD24^hi^ CD38^hi^), and as a group with potential regulatory activity (CD24^hi^ CD27^+^). Data are expressed as proportions of total B cells at each time point (Fig. 3), although note that levels of total B cells were too low 2 weeks after chemotherapy to allow analysis of these sub-compartments.

Pre-chemotherapy, CD27^+^ memory cells made up 38 % (14 % non-switched; 23 % switched) of peripheral B cells while at 3 months post-chemotherapy the B cell compartment was radically different, with only 14 % memory cells (5 % non-switched; 9 % switched; both *p* < 0.001). Interestingly, at later time-points the proportions of memory cells decreased even further and showed no sign of returning to pre-chemotherapy levels. Correspondingly, the proportions of naïve B cells increased from 54 % pre-chemotherapy to 80 % 3 months after chemotherapy (*p* < 0.001), and continued to increase reaching 85 % after 9 months. This change in B cell composition is strikingly represented as the ratio of naïve cell numbers to memory cell numbers, which was 1.31 pre-chemotherapy and increased dramatically to 8.55 at 9 months after chemotherapy (*p* < 0.001). Transitional B cells (CD24^hi^ CD38^hi^) represented 5 % of total B cells pre-chemotherapy and this proportion greatly expanded at 3 months post-chemotherapy to 23 % (*p* < 0.001), before then progressively falling, reaching 14 % by 9 months and thereby showing a trend at later time-points to return to pre-chemotherapy levels. Finally, CD24^hi^ CD27^+^ B cells, which have been reported to have a regulatory function [18], were found to be reduced as a percentage of total B cells at all time-points post-chemotherapy compared to the levels observed prior to treatment (*p* = 0.001), further emphasising the reduced expression of CD27 on B cells post-chemotherapy.

### *Chemotherapy decreases proportions of naïve and increases proportions of memory CD4*^+^*T cells*

**Fig 4:**
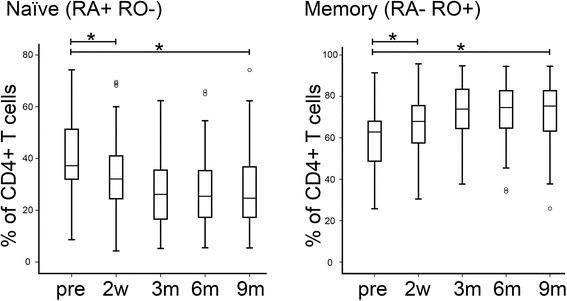
After chemotherapy CD4+ T cells comprise fewer naïve cells and more memory cells, a change that does not show a trend towards normalising even 9 months after chemotherapy. CD4+ T cell subtypes were quantified by multi-parameter flow cytometry and their numbers are presented as the proportion of the total CD4+ T cell pool. Data are shown for samples taken pre-chemotherapy (pre) and 3, 6, and 9 months (3m, 6m, 9m) after the end of chemotherapy. Boxes represent 50 % of the data, with medians (lines), interquartile ranges (whiskers) and individual outliers (circles). * *p* < 0.001

CD4^+^ T cell phenotypes were also analysed further to differentiate naïve from memory cells (Fig. 4). Naïve cells, defined as CD45RA^+^ RO^−^, made up 37 % of the entire CD4^+^ T cell pool pre-chemotherapy. This proportion was significantly reduced to 32 % 2 weeks post-chemotherapy (*p* < 0.001) and continued to fall over the course of follow up to only 25 % at 9 months post-chemotherapy. Similar significant findings were uncovered using the alternative markers CD45RA^+^ CD62L^+^ to identify naïve cells (47 % initially, falling to 35 % 2 weeks post-chemotherapy, and then 32 %, 31 % and 33 % at the same follow up time-points; *p* < 0.01 for initial levels compared to every subsequent time-point). Conversely, the proportion of memory cells, defined as CD45RA^−^ RO^+^, increased from 63 % pre-chemotherapy to 68 % 2 weeks post-chemotherapy (*p* < 0.001) and then increased progressively during follow up to 75 % at 9 months post-chemotherapy. When expressed as a ratio of naïve to memory cells within the CD4^+^ T cell compartment, the value dropped from 0.6 pre-chemotherapy to 0.47 at 2 weeks post-chemotherapy (*p* < 0.001) and continued to fall to 0.35, 0.34 and 0.33 at 3, 6 and 9 months respectively (*p* < 0.001), indicating that chemotherapy preferentially impacted on naïve cells and that this population failed to recover. Further evidence for a preferential and long-term influence on immature cells was provided from analysis of levels of positive expression of CD31 on CD45RA^+^ CD4^+^ T cells; this has been proposed as a marker of recent thymic emigrants [19, 20]. These cells decreased from 22 % of the CD4^+^ T cell pool pre-chemotherapy to 18 % at 2 weeks post-chemotherapy (*p* < 0.001), and continued to fall to 12 % after 9 months (*p* < 0.001) (Additional file 2: Figure S1).

### *Recovery of CD4*^+^*T cells correlates with recovery of switched memory B cells*

CD4^+^ T cells have a key role in B cell activation and maturation by stimulating class switching. Therefore, we next examined whether levels of CD4^+^ T cells were associated with different compositions of the B cell pool before chemotherapy or during recovery. No correlations were evident pre-chemotherapy. However, correlations were evident during reconstitution of the lymphocyte pool from 3 months to 9 months post-chemotherapy. Absolute numbers of CD4^+^ T cells correlated significantly with absolute numbers of switched memory B cells at all three time points (3, 6 and 9 months: coefficient 0.22 [*p* < 0.05], 0.34 [*p* = 0.002] and 0.32 [*p* = 0.005]) and this was reflected in a negative correlation with the naïve to memory ratio within the B cell pool (3, 6 and 9 months: coefficient -0.21 [*p* < 0.05], -0.29 [*p* = 0.01] and -0.24 [*p* = 0.035]).

### Differences in chemotherapy regimen influence depletion and repopulation dynamics

Next, we examined whether any clinico-pathological factors were significantly associated with differences in chemotherapy response of B or CD4^+^ T lymphocytes. Factors tested included patient age, smoking status, tumour size, tumour grade, lymph node status, hormone receptor status, radiotherapy treatment and chemotherapy regimen. Correlations identified are described below.

**Fig 5:**
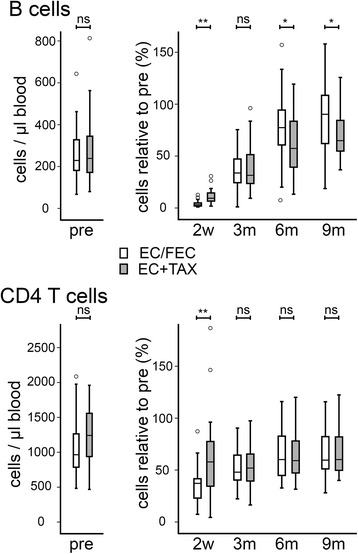
Differences in chemotherapy regimen correlate with the extent of B cell and CD4+ T cell depletion and with the dynamics of B cell recovery. Patients received anthracycline-based chemotherapy (EC/FEC, open boxes; *n* = 39) or anthracyclines followed by taxanes (EC + TAX, filled boxes; *n* = 44). Peripheral B cell and CD4+ T cells were quantified by multi-parameter flow cytometry either before chemotherapy (pre) or at time-points after the end of chemotherapy (2 weeks [2w]; 3, 6 or 9 months [3m, 6m, 9m]). Data are shown as absolute counts (left panels) or relative to the matched pre-chemotherapy level (right panels). Boxes represent 50 % of the data, with medians (lines), interquartile ranges (whiskers) and individual outliers (circles). *p < =0.05 **p < 0.01

We found that variations in chemotherapy regimens had significant influences on both B and CD4^+^ T cells (Fig. 5). Patients within our cohort were treated with anthracycline-based regimens (epirubicin and cyclophosphamide [EC], or 5-flurouracil, epirubicin and cyclophosphamide [FEC]) for 6 cycles, or alternatively were treated with 2 EC cycles followed by further cycles of the taxane, docetaxel [EC + TAX]. It should be noted that patients who received docetaxel also received Granulocyte Colony Stimulating Factor (G-CSF) in order to combat neutropenia [7]. The patients in the EC/FEC and the EC + TAX groups did not significantly differ in their pre-chemotherapy levels of either B or CD4^+^ T cells (Fig. 5, left panels). However, patients receiving EC/FEC showed significantly greater B cell depletion compared to the EC + TAX group at 2 weeks after treatment (depleted to 3 % vs 8 % respectively; *p* < 0.001). Surprisingly, the EC/FEC group, who suffered the greatest depletion, recovered B cells significantly more quickly than the EC + TAX group, with the difference between the groups reversed and significant at both 6 months (*p* = 0.04) and 9 months (*p* = 0.03). At this 9 months time-point, the levels in the EC/FEC group were no longer significantly different from before chemotherapy (*p* = 0.248) and can be regarded as having recovered completely, while levels in the EC + TAX group remained significantly depressed (64 % of pre-chemotherapy levels; *p* < 0.001). Similarly to B cells, CD4^+^ T cells were also depleted more severely in the EC/FEC group (37 % vs 59 %; *p* = 0.002), however, by contrast with B cells, CD4^+^ T cell recovery did not differ between the groups.

### Patients who smoke reconstitute B cells more slowly and have more CD4^+^ T cells pre-chemotherapy

**Fig 6:**
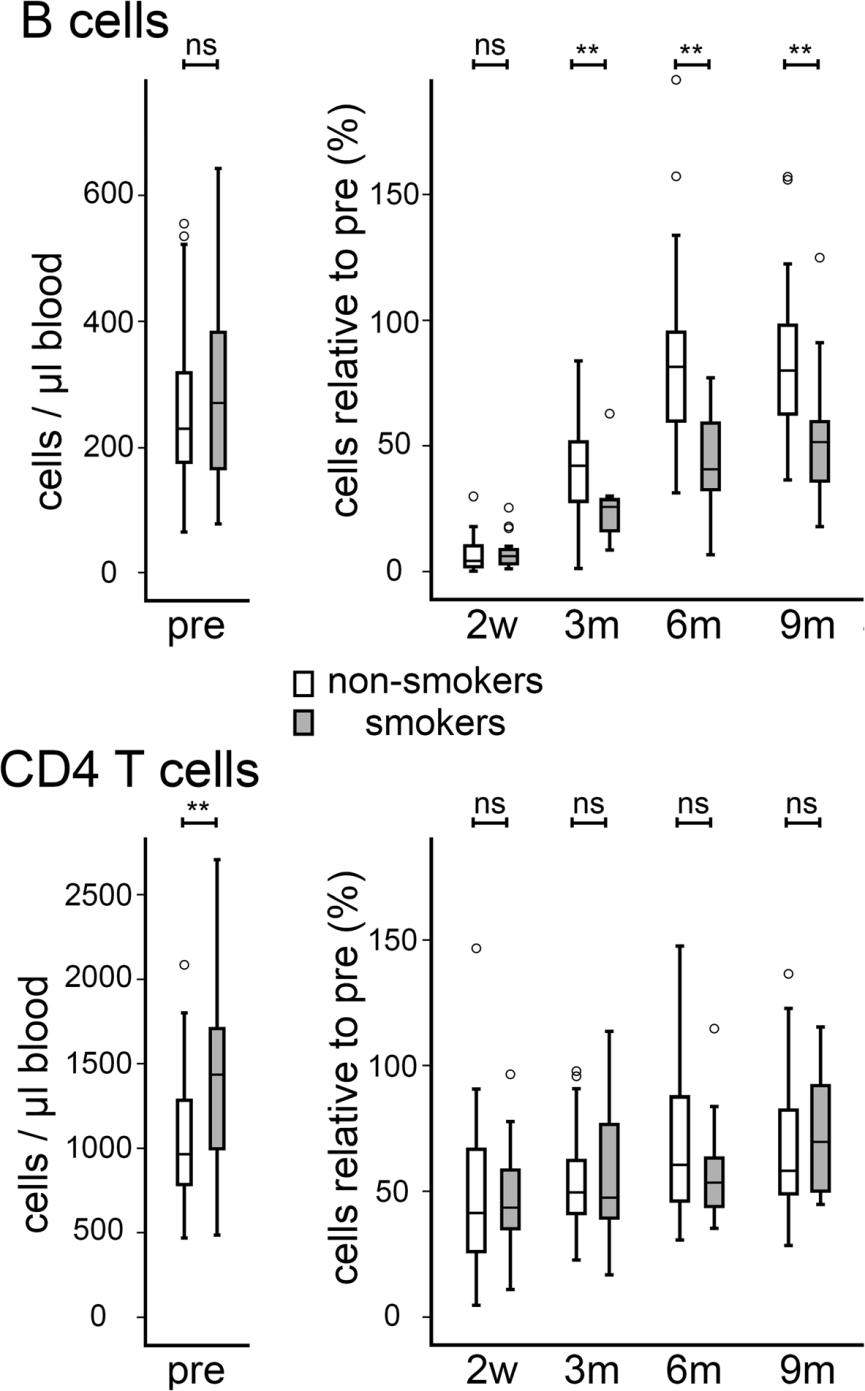
Smoking impacts on lymphocyte subtypes and their response to chemotherapy. Peripheral B cell and CD4+ T cells were quantified by multi-parameter flow cytometry either before chemotherapy (pre) or at time-points after the end of chemotherapy (2 weeks [2w]; 3, 6 or 9 months [3m, 6m, 9m]). Patients were divided into non-smokers (open boxes; *n* = 63) or smokers (filled boxes; *n* = 25). Data are shown as absolute counts (left panels) or relative to the matched pre-chemotherapy level (right panels). Boxes represent 50 % of the data, with medians (lines), interquartile ranges (whiskers) and individual outliers (circles). *p < =0.05 **p < 0.01

A further factor that influenced lymphocyte levels was smoking. Smokers and non-smokers had similar levels of B cells before chemotherapy, however recovery was substantially and significantly impaired in the smokers (Fig. 6, top panels), reaching only 51 % of pre-chemotherapy levels after 9 months as compared to 80 % in the non-smokers (*p* = 0.005). With respect to CD4^+^ T cells, smokers had significantly raised levels before chemotherapy (*p* = 0.008), but smoking did not appear to impact on the dynamics of CD4^+^ T cell repopulation (Fig. 6).

### Chemotherapy reduces serum pneumococcal and tetanus antibody titres

Given the profound changes in levels and phenotypes of B and CD4^+^ T cells, we next investigated whether circulating antibody levels were also altered and thereby that antibody-mediated immunity might potentially be affected. We examined titres of antibodies against pneumococcal and tetanus antigens, since immuno-reactivity against these antigens is typical within UK populations because of national vaccination programmes.

**Fig 7:**
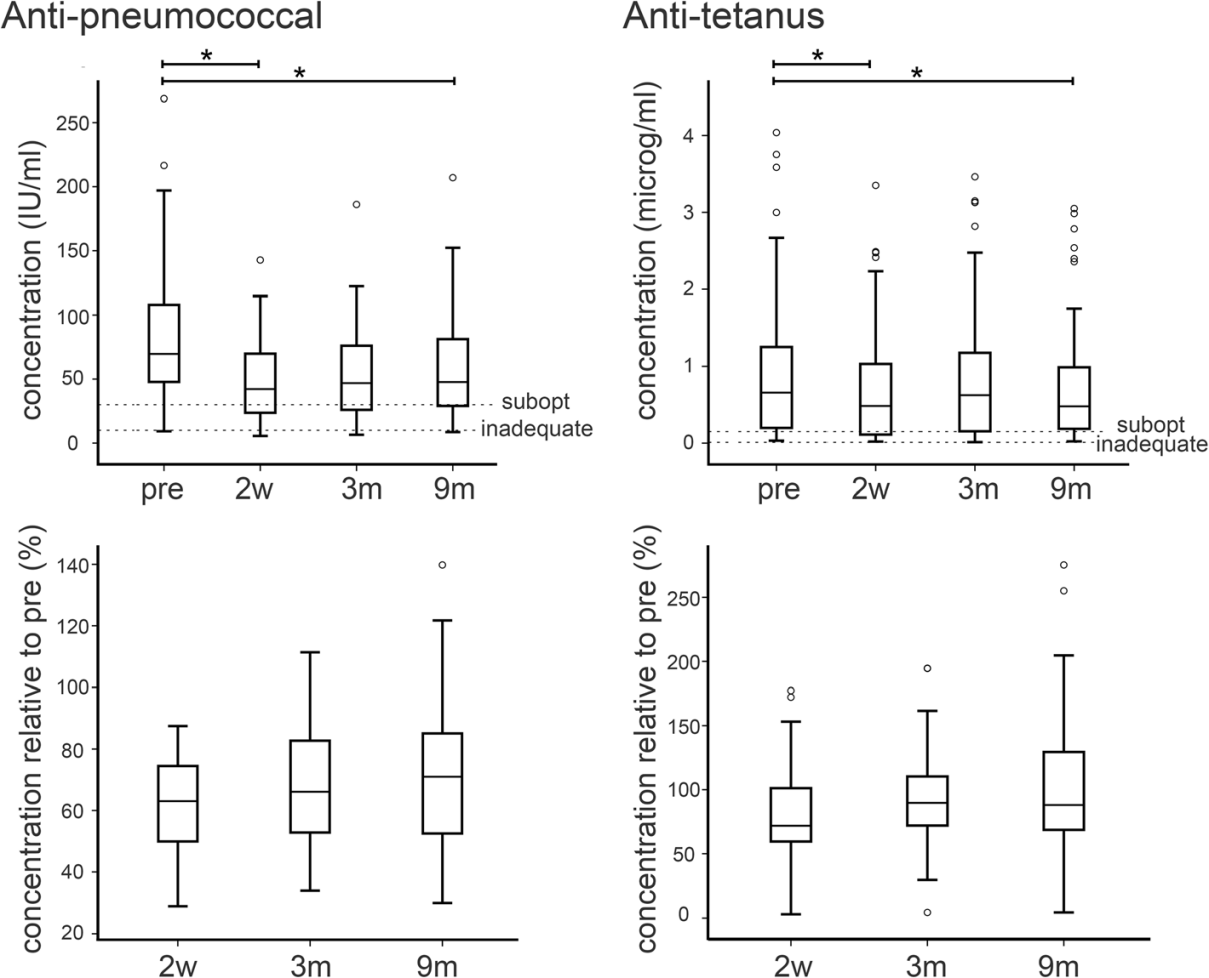
Antibodies against specific antigens are depleted and recover incompletely after chemotherapy. Antibody titres were determined in peripheral blood samples taken from breast cancer patients either before chemotherapy (pre) or at various time-points after the end of chemotherapy (2 weeks [2w]; 3 or 9 months [3m, 6m, 9m]). Data are shown as serum concentrations (top panels) or relative to the matched pre-chemotherapy level (bottom panels). Boxes represent 50 % of the data, with medians (lines), interquartile ranges (whiskers) and individual outliers (circles). * *p* < 0.001

Firstly, we noted that titres pre-chemotherapy within our patient cohort were relatively low overall, with some patients showing titres that would be clinically regarded as offering insufficient protection against the infectious agents (15 % of patients were defined as “suboptimal” and 2 % “inadequate” for anti-pneumococcus; 21 % “suboptimal” for anti-tetanus; Fig. 7 top panels). This may reflect reduced protection associated with older age [21] and/or an extended time period after vaccination, or it may be that some patients had not had been vaccinated at all. Nevertheless, chemotherapy was associated with significant changes in titres (Fig. 7), even on the background of low initial levels. At 2 weeks post-chemotherapy, pneumococcal antibody levels were lower than the pre-chemotherapy level in all patients tested (*p* < 0.001), and they remained significantly lower than pre-chemotherapy levels overall at 3 and 9 months (*p* < 0.001). At 9 months numbers of patients with levels defined as suboptimal (19 %) or inadequate (6 %) were both increased as compared to pre-chemotherapy. Levels of tetanus antibodies were also significantly reduced 2 weeks post-chemotherapy (*p* = 0.001) and remained so for the whole study period (*p* < 0.05), although the reduction was less striking than for anti-pneumococcus. In this case the proportions of patients with suboptimal or inadequate levels of antibody did not differ between 9 months and pre-chemotherapy.

Levels of both antibodies did not correlate with levels of total B cells, the levels of the B cell subtypes described previously or with chemotherapy regimen. Antibody levels did, however, show some correlations with smoking. Anti-pneumococcal antibody levels were significantly higher before chemotherapy in non-smokers as compared to smokers (*p* < 0.05) although this difference did not maintain significance after chemotherapy. By contrast, anti-tetanus antibody levels were not significantly different in these groups pre-chemotherapy, but recovery of levels was significantly impaired in smokers when assessed as a proportion of initial levels, with smokers reaching only 72 % of initial levels as compared to 94 % in non-smokers (*p* < 0.02). In the smokers, these levels were still significantly depressed (*p* = 0.03), while in the non-smokers they were no longer significantly different from pre-chemotherapy levels.

## Discussion

Chemotherapy is the main stay of treatment for relatively poor prognosis breast cancers. Despite the routine nature of this treatment, knowledge of its impacts on the immune system is poor. While the focus of breast cancer treatment rightly remains on effective cancer cures, there is increasing recognition that other aspects of post-treatment welfare require additional attention [22, 23]. This is particularly the case because breast cancer survival times have increased greatly meaning that post-treatment complications may be suffered for many years. Moreover, there is increasing evidence that competent immune function is involved with response to adjuvant biologic treatments such as trastuzumab [24], raising the possibility that chemotherapy-induced immune dysfunction could render patients less responsive to modern targeted therapies. Similarly, host immune function is critical for a wide range of immunotherapies that are in development [25], and careful consideration of sequencing may be required if these are to be combined with cytotoxic chemotherapies. Knowledge of what impacts chemotherapy has on the immune system may be key to using these immunotherapies effectively.

We observed that chemotherapy caused short-term depletion of all main subtypes of circulating lymphocytes (3-6 months), and prolonged (>9 months) depletion of B and CD4^+^ T cells (Fig. 1). This is compatible with a previous smaller study showing sustained depletion of CD4^+^, but not CD8^+^, T cells after FEC chemotherapy for breast cancer, although unfortunately B cell were not studied in this case [26]. We also analysed the phenotype of the reconstituting B and T lymphocytes in detail. Post-chemotherapy, the B cell compartment contained an increased proportion of naïve (CD27^−^) cells and fewer memory cells (CD27^+^), affecting both non-switched (marginal zone) and switched (via germinal centre reactions) memory B cells (Fig. 3), with an increased proportion of transitional CD24^hi^ CD38^hi^) B cells. CD4^+^ T phenotypes showed the reverse switch, with more memory (CD45^RO+^) and fewer naïve cells (CD45^RA+^) (Fig. 4). For CD4^+^ T cells, this change has been reported previously in breast cancer patients [26], although we have expanded the observation to recent thymic emigrants, while for B cells, this is – to our knowledge – a new observation. Relevant parallels may be drawn with lymphocyte repopulation following depletion by antibody or conditioning regimens prior to hematopoietic stem cell transplantation (HSCT). In these contexts, repopulating B cells are also predominantly naïve with an expansion of the transitional compartment, whilst surviving CD4^+^ T cells are skewed towards a memory phenotype [27–29]. A difference, however, is that we did not see evidence of substantially elevated (‘rebound’) numbers of B cells during recovery, as has been reported following HSCT [30].

Recovery post-HSCT may also inform consideration of the longer-term immune prospects of breast cancer patients post-chemotherapy with regards to susceptibility to infections. HSCT patients suffer lymphopenia in the months following transplantation and are at high risk of viral reactivations, typically earlier in recovery [31], and are more prone to bacterial infections later, especially in the case of delayed B cell reconstitution [32, 33]. Similarly, viral reactivation is relatively common in breast cancer patients during or shortly after chemotherapy [34, 35], although this has not been related to depleted lymphocyte levels. Of relevance also is that treatment with alemtuzumab is not associated with increased susceptibility to infections and patients have normal antibodies levels and vaccine responses [36, 37], although recent reports suggest treatment-related autoimmunity, particularly affecting thyroid and kidney [38].

We analysed antibody titres as a potential readout of the functional impact of lymphocyte depletion and determined that both anti-pneumococcal and anti-tetanus antibody levels were significantly reduced for at least 9 months post-chemotherapy (Fig. 7), leading to an increased proportion of patients clinically regarded as lacking appropriate protection against these antigens. Sustained repression of these antibody levels did not correlate with low levels of any specific lymphocyte subtype, therefore it seems likely that reduced serum antibodies related to loss of bone marrow resident plasma cells or long-lived memory B cells. In the context of breast cancer we believe these are novel observations, but loss of humoral immunity to viral antigens after chemotherapy has previously been reported in children [39, 40]. In addition, this disconnect between B cell numbers/phenotype and serological measures of antibody protective immunity is well established in the context of bone marrow transplantation studies [29].

We also demonstrated for the first time that the depletion extent and recovery dynamics of B and CD4^+^ T cells are influenced by factors that are potentially under control of oncologists (chemotherapy regimen) or patients (smoking) (Figs. 5 and 6). With respect to chemotherapy, regimens of repeated anthracycline cycles were significantly more damaging to B and CD4^+^ T cells than those using anthracyclines followed by taxanes. Surprisingly, while the latter combination spared B cells in the short-term, it was associated with significantly and substantially reduced longer-term recovery. Whether these differences relate to the cytotoxics themselves, or to the G-CSF given with taxanes is uncertain. G-CSF is given after every taxane cycle as a prophylactic for neutropenia [7], but is known to mobilise hematopoietic stem cells to the periphery [41], reportedly inhibiting B cell development within the marrow [42], therefore it is plausible that it might be a substantial influence on peripheral lymphocyte levels. Smoking has previously been associated with increased levels of both peripheral B and CD4^+^ T cells [43], while we found pre-chemotherapy levels of CD4^+^ T cells, but not B cells, to be significantly higher in smokers. More interestingly, we are the first to show smoking to be associated with reduced recovery of B cell post-chemotherapy. This observation may relate to reports of smoking causing impaired B cell development in bone marrow [44] and reduced levels of peripheral hematopoietic stem cells [45]. The data on chemotherapy agents and on smoking could be interpreted to suggest that the combination of factors that gives the very worst recovery of B cells should potentially be avoided to save-guard patients’ immune health.

## Conclusion

This study has demonstrated that the adaptive immune system is altered following chemotherapy for at least 9 months post therapy. Further investigations will be required to establish whether clinical management should be modified to avoid the worst impacts on the immune system, and whether revaccination against common immunogens should be considered in some cases.

## Additional files

Additional file 1
**Table S1**. Levels of peripheral blood lymphocytes of different subtypes do not differ in breast cancer patients pre-chemotherapy from control healthy women. Absolute numbers of the lymphocyte subgroups shown were determined by multi-parameter flow cytometry on peripheral blood samples taken from breast cancer patients before chemotherapy or from control health women. Data are shown as median absolute counts with interquartile ranges (brackets). (DOC 28 kb) Additional file 2
**Figure S1**. The proportion of CD4+ T cells defined as recent thymic emigrants is reduced after chemotherapy and shows no evidence of normalising. Recent thymic emigrant CD4+ T cells were quantified by multi-parameter flow cytometry and their numbers are presented as the proportion of the total CD4+ T cell pool. Data are shown for samples taken pre-chemotherapy (pre) and 3, 6, and 9 months (3m, 6m, 9m) after the end of chemotherapy. Boxes represent 50 % of the data, with medians (lines), interquartile ranges (whiskers) and individual outliers (circles). * *p* < 0.001. (TIF 192 kb)

## Abbreviations

EC: Epirubicin and cyclophosphamide
EC + TAX: Epirubicin and cyclophosphamide, followed by docetaxel
ELISAs: Enzyme-linked immunosorbent assay
ER: Estrogen receptor
FEC: Flurouracil, epirubicin and cyclophosphamide
G-CSF: Granulocyte Colony Stimulating Factor
PR: Progesterone receptor
