# Supplementary material for: Lymphocyte depletion and repopulation after chemotherapy for primary breast cancer
Source: Breast Cancer Res. 2016 Jan 26;18:10. doi: 10.1186/s13058-015-0669-x (PMC4727393; doi:10.1186/s13058-015-0669-x)
Supplement: Additional file 1 — Table S1. Levels of peripheral blood lymphocytes of different subtypes do not differ in breast cancer patients pre-chemotherapy from control healthy women. Absolute numbers of the lymphocyte subgroups shown were determined by multi-parameter flow cytometry on peripheral blood samples taken from breast cancer patients before chemotherapy or from control health women. Data are shown as median absolute counts with interquartile ranges (brackets). (DOC 28 kb) [file 13058_2015_669_MOESM1_ESM.doc]

## Additional file 1: Table S1. Levels of peripheral blood lymphocytes of different subtypes do not differ in breast cancer patients pre-chemotherapy from control healthy women. Absolute numbers of the lymphocyte subgroups shown were determined by multi-parameter flow cytometry on peripheral blood samples taken from breast cancer patients before chemotherapy or from control health women. Data are shown as median absolute counts with interquartile ranges (brackets).

|  | **Breast cancer cases** **pre-chemotherapy**  median cells/μl (IQR) | **Control cohort** median cells/μl (IQR) | **p value** (Mann-Whitney U test) |
| --- | --- | --- | --- |
| CD4 T cells | 1034.5 (675) | 992 (262.5) | 0.262 |
| CD8 T cells | 426 (371) | 529 (347) | 0.139 |
| B cells | 243 (179) | 272 (221.5) | 0.576 |
| NK cells | 233.5 (118) | 231 (152) | 0.962 |
