# Supplementary figures and images for: Lymphocyte depletion and repopulation after chemotherapy for primary breast cancer
Source: Breast Cancer Res. 2016 Jan 26;18:10. doi: 10.1186/s13058-015-0669-x (PMC4727393; doi:10.1186/s13058-015-0669-x)

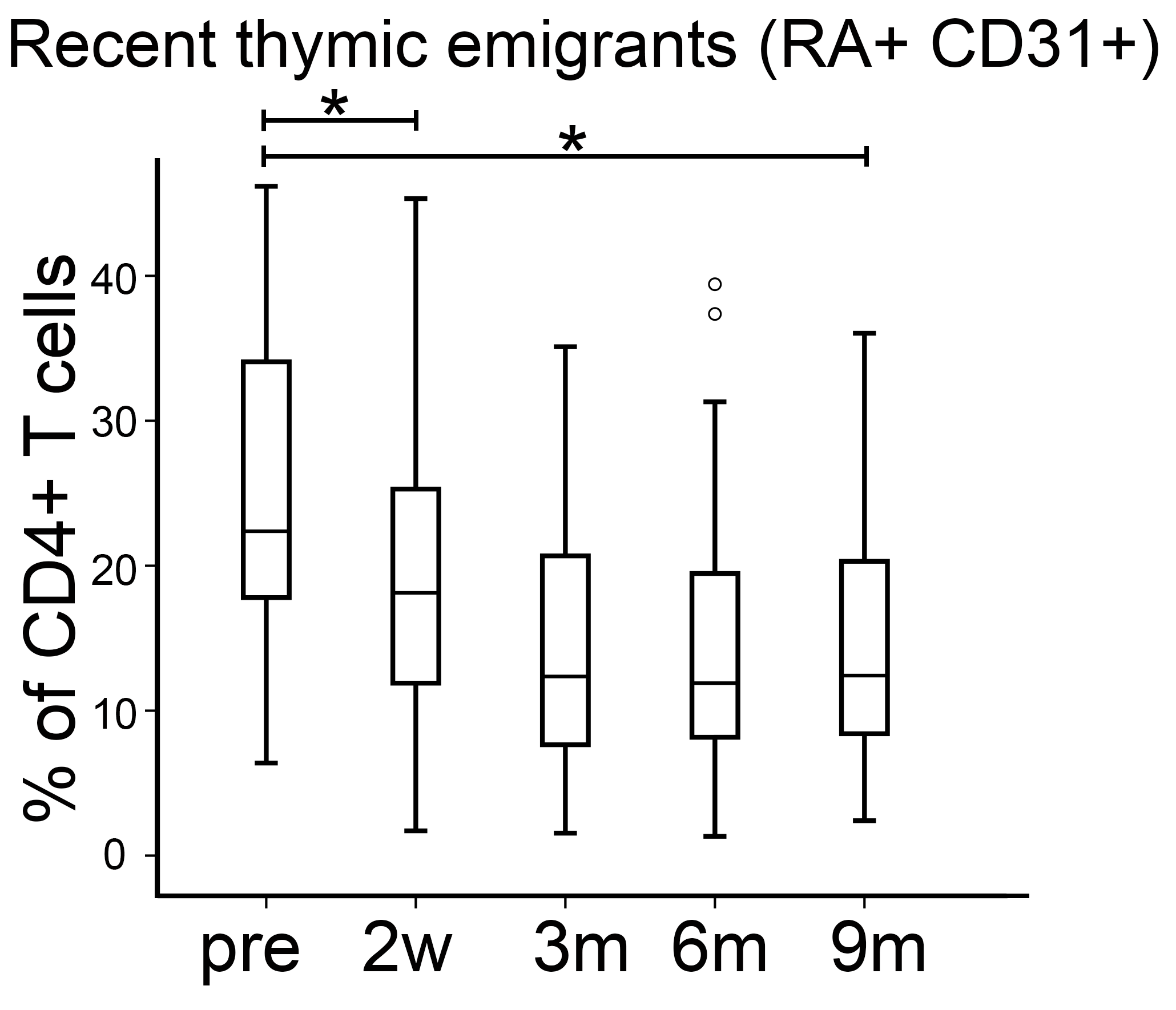

Supplement: Additional file 2 — Figure S1. The proportion of CD4+ T cells defined as recent thymic emigrants is reduced after chemotherapy and shows no evidence of normalising. Recent thymic emigrant CD4+ T cells were quantified by multi-parameter flow cytometry and their numbers are presented as the proportion of the total CD4+ T cell pool. Data are shown for samples taken pre-chemotherapy (pre) and 3, 6, and 9 months (3m, 6m, 9m) after the end of chemotherapy. Boxes represent 50 % of the data, with medians (lines), interquartile ranges (whiskers) and individual outliers (circles). * p < 0.001. (TIF 192 kb) [file 13058_2015_669_MOESM2_ESM.tif]
